# Mineralocorticoid Receptor Antagonist Pretreatment to MINIMISE Reperfusion Injury After ST‐Elevation Myocardial Infarction (The MINIMISE STEMI Trial): Rationale and Study Design

**DOI:** 10.1002/clc.22401

**Published:** 2015-05-19

**Authors:** Heerajnarain Bulluck, Georg M. Fröhlich, Shah Mohdnazri, Reto A. Gamma, John R. Davies, Gerald J. Clesham, Jeremy W. Sayer, Rajesh K. Aggarwal, Kare H. Tang, Paul A. Kelly, Rohan Jagathesan, Alamgir Kabir, Nicholas M. Robinson, Alex Sirker, Anthony Mathur, Daniel J. Blackman, Cono Ariti, Arvindra Krishnamurthy, Steven K. White, Pascal Meier, James C. Moon, John P. Greenwood, Derek J. Hausenloy

**Affiliations:** ^1^ Department of Cardiology Heart Hospital London United Kingdom; ^2^ National Institute of Health Research University College London Hospitals Biomedical Research Centre London United Kingdom; ^3^ Hatter Cardiovascular Institute Institute of Cardiovascular Science London United Kingdom; ^4^ Department of Cardiology Leeds General Infirmary Leeds United Kingdom; ^5^ Department of Cardiology Essex Cardiothoracic Center Nethermayne, Basildon United Kingdom; ^6^ London Department of Cardiology Chest Hospital London United Kingdom; ^7^ Nuffield Health Trust London United Kingdom; ^8^ London School of Hygiene and Tropical Medicine London United Kingdom; ^9^ Cardiovascular and Metabolic Disorders Program Duke‐National University of Singapore Singapore; ^10^ National Heart Research Institute Singapore National Heart Centre Singapore Singapore

## Abstract

Novel therapies capable of reducing myocardial infarct (MI) size when administered prior to reperfusion are required to prevent the onset of heart failure in ST‐segment elevation myocardial infarction (STEMI) patients treated by primary percutaneous coronary intervention (PPCI). Experimental animal studies have demonstrated that mineralocorticoid receptor antagonist (MRA) therapy administered prior to reperfusion can reduce MI size, and MRA therapy prevents adverse left ventricular (LV) remodeling in post‐MI patients with LV impairment. With these 2 benefits in mind, we hypothesize that initiating MRA therapy prior to PPCI, followed by 3 months of oral MRA therapy, will reduce MI size and prevent adverse LV remodeling in STEMI patients. The MINIMISE‐STEMI trial is a prospective, randomized, double‐blind, placebo‐controlled trial that will recruit 150 STEMI patients from four centers in the United Kingdom. Patients will be randomized to receive either an intravenous bolus of MRA therapy (potassium canrenoate 200 mg) or matching placebo prior to PPCI, followed by oral spironolactone 50 mg once daily or matching placebo for 3 months. A cardiac magnetic resonance imaging scan will be performed within 1 week of PPCI and repeated at 3 months to assess MI size and LV remodeling. Enzymatic MI size will be estimated by the 48‐hour area‐under‐the‐curve serum cardiac enzymes. The primary endpoint of the study will be MI size on the 3‐month cardiac magnetic resonance imaging scan. The MINIMISE STEMI trial will investigate whether early MRA therapy, initiated prior to reperfusion, can reduce MI size and prevent adverse post‐MI LV remodeling.

## Introduction

Coronary artery disease is one of the leading causes of death and disability worldwide,^1^ resulting in an estimated 7.3 million deaths per year.^2^ Despite major advances in the field of interventional cardiology, the mortality of ST‐segment elevation myocardial infarction (STEMI) remains high; in‐hospital mortality is approximately 5% to 6%, increasing to 7% to 18% at 1 year.^3^ In a large US registry consisting of 606 500 patients with acute myocardial infarction, heart failure (HF) was identified in 20.4% of individuals at admission, with a further 8.6% developing HF during the hospitalization itself.^4^ The onset of HF post‐STEMI is closely related to the final myocardial infarct (MI) size. For patients presenting with an acute STEMI, the most effective therapy for limiting MI size, preserving left ventricular (LV) function, and reducing the onset of HF is timely reperfusion using primary percutaneous coronary intervention (PPCI).^5^, ^6^, ^7^, ^8^

### Myocardial Reperfusion Injury as a Neglected Therapeutic Target

Although timely reperfusion is required to salvage viable myocardium in STEMI patients, the restoration of coronary blood flow in the infarct‐related artery has the potential in itself to induce cardiomyocyte death, a phenomenon that has been termed myocardial reperfusion injury and can contribute up to 50% of the final MI size.^9^ Therefore, novel therapies capable of protecting the heart from myocardial reperfusion injury may allow one to maximize the benefits of myocardial reperfusion in terms of MI size reduction. In this regard, the mineralocorticoid receptor antagonist (MRA) spironolactone may be beneficial.

### Mineralocorticoid Receptor Antagonist Therapy

Aldosterone, a mineralocorticoid receptor agonist, is synthesised primarily in the adrenal gland and is part of the renin‐angiotensin‐aldosterone system. It is known to regulate a number of pathophysiological pathways important to post‐MI HF, including inhibition of nitric oxide activity,^10^ acute endothelial dysfunction,^11^ increased vascular tone,^12^ vascular smooth muscle cell and cardiomyocyte necrosis,^13^, ^14^ myocardial hypertrophy and fibrosis,^15^, ^16^ apoptosis,^17^ and unfavorable LV remodeling.^18^ Consequently, a number of landmark clinical studies have highlighted the significant benefits of aldosterone antagonist therapy on clinical outcomes post‐MI.^19^, ^20^, ^21^

### Mineralocorticoid Receptor Antagonist Therapy to Target Myocardial Reperfusion Injury

Experimental preclinical data have demonstrated that administering MRA therapy by a clinically relevant regimen, using either intravenous potassium canrenoate (a compatible metabolite of spironolactone) or eplerenone, after a sustained episode of myocardial ischemia and 5 minutes prior to reperfusion, protected the heart against myocardial reperfusion injury and reduced MI size by 40% to 50% in murine, rat and rabbit in vivo models of MI.^22^ An earlier experimental study had reported a cardioprotective effect with the administration of eplerenone prior to the index ischemic event in the isolated perfused rat heart.^23^ A more comprehensive review of the cardioprotective effects of MRA therapy has been summarized in the review by van den Berg et al.^24^

Therefore, in this study we aim to combine the acute benefit of MRA therapy to reduce reperfusion injury and its benefit on preventing LV remodeling after 3 months of oral therapy. We hypothesize that early intravenous MRA therapy administered prior to reperfusion followed by 3 months of oral MRA therapy can reduce MI size and reduce adverse LV remodeling in STEMI patients.

## Methods

### Overall Study Design

The MINIMISE‐STEMI trial (https://clinicaltrials.gov, NCT01882179) is a proof‐of‐concept randomized clinical trial designed to investigate whether MRA therapy initiated prior to reperfusion and continued for 3 months can reduce MI size and prevent adverse LV remodeling at 3 months in STEMI patients treated by PPCI. It is a prospective, randomized, double‐blind, placebo‐controlled clinical trial that will recruit 150 patients through 4 tertiary care hospitals in the United Kingdom. The study will be conducted in accordance with the Declaration of Helsinki and has been approved by the National Research Ethics Service. All patients will provide informed written consent.

### Overall Objective

The main objective will be to investigate whether MRA therapy initiated prior to PPCI and continued for 3 months reduces MI size and prevents adverse LV remodeling in STEMI patients.

### Study Protocol

**Figure 1:**
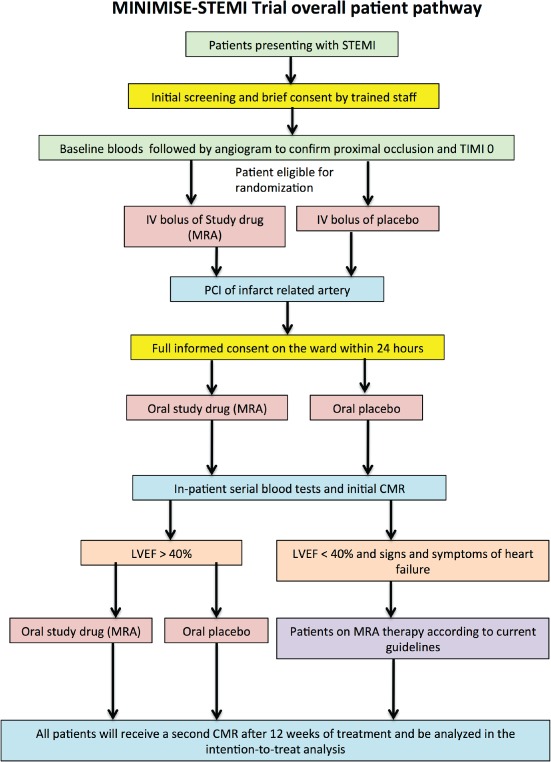
Overall patient pathway. Abbreviations: CMR, cardiac magnetic resonance imaging; IV, intravenous; LVEF, left ventricular ejection fraction; MRA, mineralocorticoid receptor antagonist; PCI, percutaneous coronary intervention; STEMI, ST‐segment elevation myocardial infarction; TIMI, Thrombolysis In Myocardial Infarction.

On immediate arrival at the hospital, patients presenting with an acute STEMI will be asked to give brief informed written consent to enter the MINIMISE‐STEMI trial and basic eligibility will be assessed. At coronary angiography, eligibility of inclusion into the trial (ie, proximal coronary artery occlusion and serum potassium (K^+^) <5.0 mmol/L and estimated glomerular filtration rate (eGFR) >30 mL/min/1.73m^2^) will be confirmed and patients will then be randomized to receive either MRA therapy or matching placebo. A detailed informed written consent will then be obtained on the ward following the PPCI procedure, within 24 hours after obtaining brief informed written consent, but before the patient is started on oral study medication. Blood tests will be taken to measure high‐sensitivity troponin T (hsTropT) and creatine kinase‐MB isoenzyme (CK‐MB) at the following time points pre‐PPCI and 6, 12, 24, and 48 hours post‐PPCI. In the first week following hospital admission, the patient will undergo a cardiovascular magnetic resonance imaging (MRI) scan (cardiovascular magnetic resonance [CMR], and this will be repeated 3 months later, after which the study ends. The overall study protocol is provided in the Figure 1.

### Patient Eligibility Criteria

All consecutive patients presenting with presumed acute STEMI in the 4 participating centers will be screened for eligibility. The inclusion and exclusion criteria are listed in Table 1.

**Table 1:** Patient Inclusion and Exclusion Criteria

Inclusion criteria for entry into trial (assessed on arrival at hospital)
Patients age >18 years
Patients presenting with acute STEMI and eligible for PPCI (as assessed by 12‐lead ECG; ST‐segment elevation ≥2 mm [0.2 mV] in ≥2 contiguous precordial leads or ≥1mm [0.1 mm] in ≥2 adjacent limb leads)
Presentation within 12 hours after symptom onset
Inclusion criteria for randomization (assessed in cardiac catheterization laboratory)
Angiographically proven proximal occlusion (TIMI: 0) of a major coronary vessel (LAD, LCX, RCA)
Normal serum K^+^ (<5.0 mmol/L)
Exclusion criteria for entry into trial (assessed on arrival at hospital)
Patients with known LVEF ≤40%
Participation in another trial
Cardiogenic shock (positive shock index OR need for catecholamine support OR SBP <90 mm Hg)
Killip class >2
Prior MI
Known compromised renal function (eGFR <30 mL/min/1.73 m^2^) or K^+^ >5.0 mmol/L
Current treatment with MRAs
Current treatment with cyclosporine
Pregnant or lactating females
Females of childbearing potential and males must be willing to use an effective method of contraception (hormonal or barrier method of birth control; abstinence) from the time consent is signed until visit 5, as per the guidance in the patient information leaflet
Allergies to IMP or its excipients
Known contraindication to CMR such as significant claustrophobia, severe allergy to gadolinium chelate contrast, presence of CMR‐contraindicated implanted devices (eg, pacemaker, ICD, CRT device, cochlear implant), imbedded metal objects (eg, shrapnel), or any other contraindication for CMR
Once the current sCr is known, patients with severely compromised renal function (eGFR <30 mL/min/1.73 m^2^) will also be excluded
Patients with known porphyria
Patients with significant liver dysfunction (INR >2)
Patients with known contraindications to treatment with spironolactone

Abbreviations: CMR, cardiovascular magnetic resonance imaging; CRT, cardiac resynchronization therapy; ECG, electrocardiogram; eGFR, estimated glomerular filtration rate; ICD, implantable cardioverter‐defibrillator; IMP, investigational medicine product; INR, international normalized ratio; K^+^, potassium; LAD, left anterior descending coronary artery; LCX, left circumflex coronary artery; LVEF, left ventricular ejection fraction; MI, myocardial infarction; MRA, mineralocorticoid receptor antagonist; PPCI, primary percutaneous coronary intervention; RCA, right coronary artery; SBP, systolic blood pressure; sCr, serum creatinine; STEMI, ST‐elevation myocardial infarction; TIMI, Thrombolysis In Myocardial Infarction.

### Randomization and Treatment Allocation

Eligible patients will be randomized via a Web‐based service (https://www.sealedenvelope.com) in a 1:1 manner to either MRA therapy or matching placebo. Randomization will be stratified by recruiting site. The patient, interventional cardiologist, caregivers, and data collector will be blinded to the treatment allocation. The study drug or placebo will be administered by the nonblinded research investigator.

### Study Drug and Placebo

#### MRA Therapy

Patients randomized to MRA therapy will receive an intravenous bolus of potassium canrenoate (200 mg, in keeping with the dose used initially by Hayashi et al^25^ and subsequently in the Aldosterone Blockade Early After Acute Myocardial Infarction [ALBATROSS] trial)^26^ followed by oral spironolactone 25 mg once daily for 2 weeks and then 50 mg once daily for the remaining 10 weeks, after which the MRA therapy will be stopped. Serum K^+^ will be checked at 2 and 4 weeks. Spironolactone will only be uptitrated to 50 mg after 2 weeks if the serum K^+^ is ≤5.0 mmol/L. If the serum K^+^ is 5.1 to 5.5 mmol/L, 25 mg spironolactone will be maintained, and if the K^+^ is >5.5 mmol/L, spironolactone will be discontinued.

#### Matching Placebo

Patients randomized to control will receive an intravenous bolus of normal saline followed by placebo control tablets for the 3 months, after which the placebo will be stopped. Serum K^+^ will be checked at 2 and 4 weeks.

### Optimal Heart Failure Treatment

The study protocol will ensure optimal medical therapy for all study participants according to current practice guidelines.^3^, ^27^, ^28^, ^29^ Therefore, patients with a left ventricular ejection fraction (LVEF) ≤40% on the initial CMR scan and evidence of HF or who have diabetes mellitus (DM) will be started on open‐label eplerenone according to current practice guidelines. These patients will be included in the intention‐to‐treat analysis.

### Cardiac Magnetic Resonance Protocol and Analysis

Each patient recruited into the MINIMISE‐STEMI trial will have 2 CMR scans, the first being performed in the week following hospital admission and the second performed at 3 months. Training in the CMR protocol will be provided to each recruiting site. All CMR scans will be analyzed by an independent MRI CoreLab at University College London.

The primary endpoint of MINMISE‐STEMI trial will be MI size on the 3‐month CMR scan (measured as the mass of late gadolinium enhancement (LGE) and expressed as a percentage of the LV mass). The CMR parameters that will be determined for the first CMR scan:

1. LVEF and indexed LV end‐systolic and end‐diastolic volumes and mass using short‐axis steady‐state free‐precession cine imaging.
2. MI size, measured by the mass of LGE (10 minutes after administration of contrast) on CMR expressed as a percentage of LV mass.
3. Area at risk (AAR), measured as the increase in T2 signal on‐T2 weighted images or T2 mapping, both of which have been validated against conventional measures of the AAR.^30^, ^31^, ^32^ The AAR will also be estimated using the modified Bypass Angioplasty Revascularization Investigation Myocardial Jeopardy Index (BARI) and Alberta Provincial Project for Outcome Assessment in Coronary Heart Disease (APPROACH) angiography scores.^33^
4. Myocardial salvage index = AAR − MI size/AAR. The myocardial salvage index (using T2‐weighted CMR and LGE) has been demonstrated to predict prognosis post‐PPCI.^34^
5. The incidence and extent of microvascular obstruction (hypo‐enhancement on LGE 20 minutes after administration of contrast).
6. The incidence and extent of intramyocardial hemorrhage (hypo‐enhancement on Siemens T2* mapping sequence).

The CMR parameters that will be determined for the second CMR scan, at 3 months:

1. LVEF and indexed LV end systolic and diastolic volumes, wall thickness, and mass using short‐axissteady‐state free‐precession cine imaging.
2. Final MI size, which represents the primary endpoint, measured by the mass of LGE and expressed as a percentage of the LV mass.

The CMR images will be analyzed by 2 experienced operators blinded to the treatment allocation and clinical outcome. Datasets will be reported in consensus, and advice from a third MRI operator will be sought in cases of disagreement (>5 g difference in infarct size between operators based on the limits of agreement for interobserver variability with the Otsu automated thresholding technique from Vermes et al).^35^ Left ventricular volumes and mass measurements will be calculated conventionally using dedicated software (CVI42 software, version 5.0.3; Circle Cardiovascular Imaging, Calgary, AB, Canada), with papillary muscles considered as part of the LV myocardium. Analysis of MI size and extent of myocardial edema will be performed using an in‐house macro written in ImageJ.^36^ The epicardial and endocardial borders will be manually traced and the areas of LGE will be quantified using the semiautomated Otsu detection method, as previously validated by Vermes et al.^35^ Any dark areas of hypo‐enhancement (microvascular obstruction ± intramyocardial hemorrhage) within the area of LGE will be included in the infarct area.^37^

### Study Endpoints

The primary endpoint of the MINIMISE‐STEMI study will be MI size quantified by LGE cardiac MRI performed at 3 months following hospital admission. The secondary endpoints are listed in Table 2. The safety endpoints will include major adverse cardiac events and side effects from MRA therapy (cardiovascular death, nonfatal MI, revascularization, hospitalization for HF, hyperkalemia, deterioration of kidney function, and need for dialysis; Table 3). Major adverse cardiac events will be reported after 3 months.

**Table 2:** Secondary Outcome Measures

Markers of myocardial reperfusion injury (myocardial blush grade, TIMI flow post‐PPCI, ST‐segment resolution at 90 minutes post‐PPCI)
Microvascular obstruction on CMR
Myocardial salvage (AAR by T2 imaging − final infarct size)
Acute myocardial infarct size (serum biomarkers and CMR on day 2–7)
Serum biomarkers (hsTropT, CK‐MB) at the following time points; 0, 6, 12, 24, and 48 hours post‐PCI (±1 hour)
LV remodeling on 3‐month CMR scan (index LVEDV and LVESV, LVEF, and LV mass and wall thickness)
Clinical outcome measures: CV death, nonfatal MI, revascularization, hospitalization for HF, hyperkalemia, deterioration of kidney function, need for dialysis

Abbreviations: AAR, area at risk; CK‐MB, creatine kinase MB isoenzyme; CMR, cardiovascular magnetic resonance imaging; CV, cardiovascular; HF, heart failure; hsTropT, high‐sensitivity troponin T; LV, left ventricular; LVEDV, left ventricular end‐diastolic volume; LVEF, left ventricular ejection fraction; LVESV, left ventricular end‐systolic volume; MI, myocardial infarction; PPCI, primary percutaneous coronary intervention; TIMI, Thrombolysis In Myocardial Infarction.

**Table 3:** Definitions of Safety Endpoints

CV death	Death due to a known CV cause or where the cause of death is unknown (ie, where no other cause of death has been identified from the medical history or an autopsy)
Nonfatal MI	Detection of rise and or fall of cardiac biomarkers with ≥1 value >99th percentile of the upper reference limit together with evidence of myocardial ischemia with ≥1 of the following: symptoms of ischemia; new ST‐T changes or new LBBB or development of pathological Q waves on the ECG; imaging evidence of new loss of viable myocardium or new regional wall‐motion abnormality
Revascularization	Any repeat PCI or CABG with or without valve within the first year postsurgery
Hospitalization for HF	Hospital admission of ≥24‐hour stay. HF will be judged to be present on symptoms (≥1 of the following: new or worsening dyspnea, orthopnea, or paroxysmal nocturnal dyspnea, or increasing fatigue/worsening exercise tolerance) and signs (1 of the following: new pulmonary edema by chest X‐ray in the absence of a noncardiac cause, crepitations believed to be due to pulmonary edema, and use of loop diuretics to treat presumed pulmonary congestion).
Hyperkalemia	Serum K^+^ level >5.5 mmol/L
Deterioration of renal function	sCr increase of >25% from baseline
Stroke	Stroke will be defined as a focal, central neurological deficit lasting >72 hours that results in irreversible brain damage or body impairment.

Abbreviations: CABG, coronary artery bypass grafting; CV, cardiovascular; ECG, electrocardiography; HF, heart failure; K^+^, potassium; LBBB, left bundle branch block; MI, myocardial infarction; PCI, percutaneous coronary intervention; sCr, serum creatinine.

### Statistical Analysis

A sample size of 50 in each group will have 80% power to detect a difference in means of 8.0 g infarct mass as assessed by MRI (the difference between a Group 1 mean, μ_1_, of 40.0 g, and a Group 2 mean, μ_2_, of 32.0 g), assuming that the common SD is 14.0 g infarct mass^38^ using a 2‐group *t* test with a 0.05 2‐sided significance level. However, we anticipate that about 10% of patients in the control group will have LVEF ≤40%, in which case they will receive the trial treatment as a matter of standard care. To allow for this dilution of the treatment effect, we have increased the sample size in each group to 62. If we further assume an attrition rate of approximately 15%, then 75 patients per group will be needed, for a total trial size of 150 patients.

Statistical analysis will be performed using SAS version 9.3 (SAS Inc., Cary, NC). The Fisher exact test will be used to compare categorical variables. Continuous variables will be compared with a mixed‐effects model to account for repeated measures. Primary data analysis will be performed in the intention‐to‐treat population, regardless of the treatment that was actually received. The results of as‐treated analyses for primary and secondary endpoints will also be calculated. Time‐to‐event analyses (secondary clinical endpoints), based on all available follow‐up data, will be performed with the use of Kaplan‐Meier estimates and be compared between groups with the use of the log‐rank test. A generalized linear model will be used to calculate risk ratios in the subgroup analyses and to test for interactions. After 75 patients have completed the trial, an interim analysis will be performed by the study statistician for safety reasons. The results will be evaluated by the data‐monitoring committee.

### Data Management, Funding and Logistics

University College London is the sponsor of the trial. Data will be collected by a paper case report form and entered onto the Web‐based electronic RedCap database. An independent data‐monitoring committee (IDMC) will be installed to monitor the progress of the study as well as any safety concerns. All expected or unexpected adverse events will be reviewed continuously by the investigators, the sponsor and the IDMC according to the sponsor's regulations. The MINIMISE‐STEMI trial is funded by the Rosetrees Trust and the National Institute for Health Research (NIHR) Clinical Research Network.

### Study Timeline

Although the study has been open to recruitment since November 2013, recruitment has been slow, but it is expected to improve with the recent expansion to a fourth center. We currently have 24 patients enrolled in the study, and we anticipate to recruit a further 51 patients over the next 6 months (July 2015), when an interim analysis will be performed. We plan to complete recruitment in February 2016, with the last patient having the 3‐month CMR scan in May 2016 and the results of the study available in August 2016.

## Discussion

The MINIMISE‐STEMI trial is designed to investigate whether MRA therapy using spironolactone initiated prior to reperfusion and continued for 3 months can reduce MI size and prevent adverse LV remodeling at 3 months in STEMI patients treated by PPCI, when compared with placebo control. The Eplerenone Post‐AMI Heart Failure Efficacy and Survival (EPHESUS) study demonstrated a 15% relative risk reduction in death at 16 months in AMI patients with LVEF <40% and HF signs administered oral eplerenone therapy (initiated 3–14 days post‐MI) when compared with matching placebo.^19^ The beneficial effects of oral MRA therapy have been confirmed in subsequent clinical trials.^20^, ^21^, ^39^ The American^27^ and European^29^ guidelines now recommend eplerenone for all patients presenting with an AMI, LV dysfunction (LVEF ≤40%), and HF symptoms or DM. Therefore, it is anticipated that about 10% of the trial participants will meet these criteria and will go on open‐label MRA therapy, and this has been taken into account in the sample‐size calculation.

Hayashi et al^25^ investigated the early use of spironolactone immediately post percutaneous transluminal coronary angioplasty in patients with anterior STEMI and showed a reduction in postinfarct LV remodeling when used in combination with an angiotensin‐converting enzyme inhibitor. Recently, the Early Eplerenone Treatment in Patients With Acute ST‐Elevation Myocardial Infarction Without Heart Failure (REMINDER) trial investigated the effect of initiating oral eplerenone therapy 12 to 24 hours following STEMI admission (in the absence of HF) and reduced the incidence of patients with elevated serum brain natriuretic peptide/N‐terminal‐pro‐brain natriuretic peptide at ≥1 month after randomization, when compared with placebo.^40^ The ALBATROSS trial is investigating the effect of MRA therapy initiated within 72 hours of symptom onset in patients with either non–ST‐segment elevation myocardial infarction or STEMI (regardless of HF status) on the 6‐month primary combined endpoint of death, resuscitated cardiac arrest, significant ventricular arrhythmia, class IA indication for an implantable defibrillator device, and new or worsening HF. In that study, the MRA therapy will comprise an IV bolus of potassium canrenoate (200 mg) followed by a daily 25‐mg dose of spironolactone for 6 months.^26^ This study has been completed, but the results are not yet published. All of these clinical studies have investigated the effect of MRA therapy initiated after reperfusion has taken place, and therefore only target post‐MI LV remodeling. None of these clinical studies have investigated the effect of targeting myocardial reperfusion injury with MRA therapy.

Whether early intravenous MRA therapy administered prior to reperfusion followed by 3 months of oral MRA therapy can reduce MI size and reduce adverse LV remodeling is not known and will be investigated in the MINIMISE‐STEMI trial.

## Conclusion

By administering the MRA therapy prior to reperfusion, we hope to target myocardial reperfusion injury and obtain additional benefit in terms of MI size reduction, which will hopefully add to the known beneficial effects of oral MRA therapy in terms of preventing post‐MI adverse LV remodeling.

## Supporting information

Key study participants and committee members
