# Supplementary material for: Mineralocorticoid Receptor Antagonist Pretreatment to MINIMISE Reperfusion Injury After ST‐Elevation Myocardial Infarction (The MINIMISE STEMI Trial): Rationale and Study Design
Source: Clin Cardiol. 2015 May 19;38(5):259–66. doi: 10.1002/clc.22401 (PMC4489325; doi:10.1002/clc.22401)
Supplement: Supplementary file 1 — Key study participants and committee members [file CLC-38-259-s001.pdf]

Appendix 1: Key study participants and committee members

**Chief Investigator**	Professor DJ Hausenloy	The Hatter Cardiovascular Institute,<br>University College London
**Primary sub-investigator**	Dr. GM Fröhlich	The Heart Hospital
**Research Fellow**	Dr H Bulluck	The Hatter Cardiovascular Institute,<br>University College London
**Sponsor's representative**	Ms Akinyemi	Research Office,<br>University College London
**Statistician**	Mr C. Ariti	Nuffield Health Trust<br>London School of Hygiene and Tropical Medicine
**Principle investigators**	Dr A. Sirker	The Heart Hospital
	Dr R. Gamma	Essex Cardiothoracic Center
	Prof A. Mathur	London Chest Hospital
	Prof JP Greenwood	Leeds General Infirmary
**Independent data monitoring committee**	Dr M Walker	University College Hospital
	Dr C Manisty	University College Hospital

For Peer Review
